# Discovery, Prevalence, and Persistence of Novel Circular Single-Stranded DNA Viruses in the Ctenophores *Mnemiopsis leidyi* and *Beroe ovata*

**DOI:** 10.3389/fmicb.2015.01427

**Published:** 2015-12-18

**Authors:** Mya Breitbart, Bayleigh E. Benner, Parker E. Jernigan, Karyna Rosario, Laura M. Birsa, Rachel C. Harbeitner, Sidney Fulford, Carina Graham, Anna Walters, Dawn B. Goldsmith, Stella A. Berger, Jens C. Nejstgaard

**Affiliations:** ^1^College of Marine Science, University of South Florida St. PetersburgSt. Petersburg, FL, USA; ^2^Skidaway Institute of Oceanography, University of GeorgiaSavannah, GA, USA; ^3^Department III, Leibniz-Institute of Freshwater Ecology and Inland Fisheries (IGB)Stechlin, Germany

**Keywords:** ctenophore, single-stranded DNA virus, CRESS-DNA virus, circular DNA virus, marine, gelatinous, plankton

## Abstract

Gelatinous zooplankton, such as ctenophores and jellyfish, are important components of marine and brackish ecosystems and play critical roles in aquatic biogeochemistry. As voracious predators of plankton, ctenophores have key positions in aquatic food webs and are often successful invaders when introduced to new areas. Gelatinous zooplankton have strong impacts on ecosystem services, particularly in coastal environments. However, little is known about the factors responsible for regulating population dynamics of gelatinous organisms, including biological interactions that may contribute to bloom demise. Ctenophores are known to contain specific bacterial communities and a variety of invertebrate parasites and symbionts; however, no previous studies have examined the presence of viruses in these organisms. Building upon recent studies demonstrating a diversity of single-stranded DNA viruses that encode a replication initiator protein (Rep) in aquatic invertebrates, this study explored the presence of circular, Rep-encoding single-stranded DNA (CRESS-DNA) viruses in the ctenophores *Mnemiopsis leidyi* and *Beroe ovata* collected from the Skidaway River Estuary and Savannah River in Georgia, USA. Using rolling circle amplification followed by restriction enzyme digestion, this study provides the first evidence of viruses in ctenophores. Investigation of four CRESS-DNA viruses over an 8-month period using PCR demonstrated temporal trends in viral prevalence and indicated that some of the viruses may persist in ctenophore populations throughout the year. Although future work needs to examine the ecological roles of these ctenophore-associated viruses, this study indicates that viral infection may play a role in population dynamics of gelatinous zooplankton.

## Introduction

Gelatinous zooplankton, including ctenophores and jellyfish, play critical roles in marine ecosystems (Mills, 1995; Schneider and Behrends, 1998; Brodeur et al., 2002). Global biomass of gelatinous zooplankton is estimated at 38.3 Tg carbon (Lucas et al., 2014) and these organisms often seasonally dominate planktonic biomass (Costello et al., 2012). Ctenophore and jellyfish population dynamics can significantly affect carbon cycling in marine systems since blooms of these organisms release large quantities of dissolved organic matter, diverting carbon from higher trophic levels toward rapid uptake and respiration by bacterioplankton (Condon et al., 2011). In addition, upon bloom termination/collapse, high levels of microbial respiration associated with decomposition of senescing gelatinous organisms (Tinta et al., 2010, 2012) can lead to localized oxygen depletion (Pitt et al., 2009). Recent work has also demonstrated that sinking jellyfish carcasses (i.e., the so-called “jelly-pump”) can contribute significantly to carbon and nutrient fluxes to the deep sea (Sweetman and Chapman, 2015) and attract dense communities of deep-sea scavengers that quickly consume the gelatinous biomass (Sweetman et al., 2014).

Ctenophores are voracious gelatinous predators of plankton (Colin et al., 2010) and are highly successful biological invaders when introduced to new areas due to their dietary flexibility, wide tolerance for salinity and temperature, and reproductive strategies (Costello et al., 2012; Jaspers et al., 2015). For example, the lobate ctenophore *Mnemiopsis leidyi* A. Agassiz, 1865, which is indigenous to the temperate Atlantic coasts of North and South America, including the subtropical Gulf of Mexico (Costello et al., 2012), has gained attention in the past three decades due to its widespread invasion and negative impacts on fisheries of the Aegean, Baltic, Black, Caspian, Mediterranean, and North Seas, as well as the South Pacific Ocean (Ivanov et al., 2000; Purcell et al., 2001; Bilio and Niermann, 2004; Javidpour et al., 2009; Reusch et al., 2010; Costello et al., 2012; Bolte et al., 2013). Due to their r-selected life strategies, adaptability, and feeding behavior, blooms of *M. leidyi* exert extreme predation pressure on the abundance and community composition of co-occurring plankton (Javidpour et al., 2009; Granhag et al., 2011). *M. leidyi* adults feed on mesozooplankton and larvae feed on microzooplankton (Stoecker et al., 1987; Sullivan and Gifford, 2004); the depletion of these zooplankton groups can lead to cascading effects on lower trophic levels by selecting for specific taxa that are ideal prey items for developing ctenophores (Dinasquet et al., 2012b; McNamara et al., 2013). While some native scyphozoa, such as *Cyanea capillata*, can prey upon *M. leidyi* (Hosia and Titelman, 2010), one of the most efficient predators of *M. leidyi* is another ctenophore, *Beroe ovata*. In areas where both species have invaded, *B. ovata* can effectively limit *M. leidyi* blooms, and intentional introduction of *B. ovata* has been suggested as a biological control mechanism for invasive *M. leidyi* blooms (GESAMP, 1997; Bilio and Niermann, 2004).

Investigating the associations between ctenophores and other organisms is an important step toward better understanding ctenophore ecology. Ctenophores have been shown to have a variety of invertebrate parasites and symbionts (Ohtsuka et al., 2009), including parasitic anemones (Bumann and Puls, 1996; Reitzel et al., 2007), platyhelminth worms (Yip, 1984; Martorelli, 2001), hyperiid amphipods (Sorarrain et al., 2001; Gasca and Haddock, 2004), peritrich ciliates (Duggins et al., 1989; Estes et al., 1997), rhizopod amoebae (Moss et al., 2001a), and dinoflagellates (Mills and McLean, 1991). The presence of bacteria in ctenophores was initially noted through microscopy (Moss et al., 2001b) and culture-based methods (Saeedi et al., 2013); however, a number of recent studies examining ctenophore-bacterial interactions have also indicated that ctenophores maintain unique bacterial assemblages that are distinct from the surrounding water (Moss et al., 2001b; Daniels and Breitbart, 2012; Dinasquet et al., 2012a; Hammann et al., 2015; Hao et al., 2015).

Although these recent advances have improved our understanding of microbiological associations that may play a role in ctenophore ecology, no studies have yet examined the presence of viruses in these organisms. Building upon recent studies demonstrating a diversity of single-stranded DNA viruses encoding a replication initiator protein (Rep) in a wide variety of aquatic invertebrates (Dunlap et al., 2013; Hewson et al., 2013a,b; Ng et al., 2013; Pham et al., 2014; Soffer et al., 2014; Dayaram et al., 2015; Fahsbender et al., 2015; Rosario et al., 2015), this study explored the presence of circular, Rep-encoding single-stranded DNA (CRESS-DNA) viruses in the ctenophores *M. leidyi* and *B. ovata* collected from the Skidaway River Estuary and Savannah River in coastal Georgia, USA. Four ctenophore-associated CRESS-DNA viruses were identified using rolling circle amplification followed by restriction enzyme digestion. This study reports the first viruses to be described in gelatinous zooplankton. Investigation of the prevalence and persistence of these CRESS-DNA viruses over an 8-month period indicates that some of the viruses become prevalent at certain times of the year and some may persist in ctenophore populations. Although future work needs to examine the ecological roles of these ctenophore-associated viruses, this study indicates that viral infection may be an important factor to consider when examining bloom dynamics of gelatinous zooplankton.

## Materials and methods

### Sample collection and processing

Zooplankton samples were collected between February and October 2013 from the marine-brackish Skidaway River Estuary off Skidaway Institute of Oceanography's dock and up the Savannah River entrance, between the city of Savannah and the South Atlantic Bight, coastal Georgia, USA. Samples from the dock were collected using a 500 μm mesh plankton net with a 0.5 m opening, and the Savannah River samples were collected using a 280 μm mesh plankton net with a 0.5 m opening. Two species of ctenophores (*Mnemiopsis leidyi* and *Beroe ovata*) were picked from these samples (Supplemental Table 1). Specimens were measured for length and volume and washed in autoclaved river water. For *M. leidyi*, most specimens were also dissected using sterile techniques to remove the stomach in an effort to avoid contamination from gut contents. For *B. ovata* specimens, due to their large size, only the comb rows were processed after sterile dissection. Co-occurring copepods (primarily *Acartia tonsa*) were also collected to determine the specificity of identified viruses (see below). Specimens were flash frozen in liquid nitrogen and stored at −80°C in individual 1.8 ml cryovials until shipment to the University of South Florida on dry ice.

### Viral genome discovery

Ctenophore specimens were used for discovery of CRESS-DNA viruses following a protocol previously described for marine invertebrates (Rosario et al., 2015). For this purpose, upon thawing, ctenophores were washed three times with sterile SM buffer [0.1 M NaCl, 50 mM Tris-HCl (pH 7.5), 10 mM MgSO_4_] and placed into a 1.5 ml microcentrifuge tube containing 500 μl of sterile SM buffer and 1 mm sterile glass tissue beads. These samples were homogenized for 1 min in a bead beater (Biospec Products). Five hundred microliters of sterile SM buffer were then added to the homogenized tissue, vortexed, and centrifuged at 6000 × g for 6 min. Approximately 800 μl of supernatant from each tissue homogenate were filtered through a 0.45 μm Sterivex filter (Millipore) to partially purify virus particles. DNA was extracted from 200 μl of the collected filtrate from each sample using the QIAamp MinElute Virus Spin Kit (Qiagen) according to the manufacturer's protocol. The DNA extract from each specimen was amplified via rolling circle amplification (RCA) using the Illustra TempliPhi Amplification kit (GE Healthcare), which preferentially amplifies small circular templates (Kim et al., 2008; Kim and Bae, 2011). RCA-amplified DNA was digested with the following 12 FastDigest restriction enzymes (RE): BamHI, EcoRV, PdmI, HindIII, KpnI, PstI, XhoI, SmaI, BGlII, EcoRI, XbaI, NcoI (Life Technologies) in separate reactions with the goal of obtaining complete unit-length viral genomes as described previously (e.g., Rosario et al., 2012a, 2015). RE digestions were visualized on a 1.5% agarose gel, then bands within a size range of 1000–4000 bp were excised and purified using the Zymoclean Gel DNA Recovery Kit (Zymo Research). Cleaned RCA digested products containing “sticky-ends” were cloned into pre-digested pGem-3Zf(+) vectors (Promega) with the corresponding RE. Blunt-ended products were cloned using the CloneJET PCR Cloning kit (Life Technologies). All clones were commercially Sanger sequenced by Eurofins Scientific using vector primers. Genomes exhibiting significant similarities to eukaryotic CRESS-DNA viruses as determined by BLASTx searches against GenBank (Altschul et al., 1997) were completed through primer walking with a minimum of 2x coverage.

### Genome analysis

All genomes were assembled using the Geneious software version R7 (Biomatters). Major open reading frames (ORFs) encoding putative proteins larger than 100 amino acids long were identified and annotated using SeqBuilder version 11.2.1 (Lasergene). Genomic features associated with CRESS-DNA viruses were manually identified, including a putative origin of replication (*ori*) marked by a canonical nonanucleotide motif (NANTATTAC) at the apex of a predicted stem-loop structure as well as rolling circle replication and helicase motifs characteristic of eukaryotic CRESS-DNA virus Reps (Rosario et al., 2012b). Non-Rep-encoding major ORFs were analyzed using the DisProt VL3 disorder predictor (Obradovic et al., 2003; Sickmeier et al., 2007) to identify intrinsically disordered protein (IDP) profiles that have been observed in CRESS-DNA virus capsid proteins (Rosario et al., 2015). Rep amino acid sequence pairwise identities among CRESS-DNA viruses identified in ctenophores as well as those reported from other marine organisms and a variety of marine environments were calculated using the Species Demarcation Tool (SDT) software version 1.2 (Muhire et al., 2014). Pairwise identity comparisons were summarized and displayed using heat maps produced by SDT.

### PCR for specific ctenophore viruses

The prevalence of the newly identified CRESS-DNA viral genomes was determined using a PCR assay. For this purpose, specific primers targeting the putative *rep* gene of each viral genome were designed to screen a total of 153 ctenophore specimens. Each 50 μL PCR mixture contained 1 U Apex Taq DNA polymerase (Genesee Scientific), 1X Apex ammonium reaction buffer, 1.5 mM Apex MgCl_2_, 0.5 μM concentration of each primer, 0.2 mM deoxynucleoside triphosphates (dNTPs), and 0.6 μL of RCA-amplified template DNA. PCR conditions were as follows: 94°C for 5 min, 35 cycles of [95°C for 45 s, annealing temp from Table 1 for 45 s, 72°C for 1 min], followed by an 8 min final extension at 72°C. In addition to BLAST searches of primer sequences against GenBank, several positive PCR products of the expected size (two per primer pair) were Sanger sequenced to confirm primer specificity. The same PCR assays were also used to test pooled copepod samples (*n* = 30, containing an average of 20 individuals each) homogenized and extracted in the same manner as ctenophore samples.

**Table 1:** **Primers and annealing temperatures used for PCR assays designed to investigate the prevalence of ctenophore-associated circular viruses (CtaCVs)**.

**Virus name**	**Primer sequences (5′–3′)**	**Annealing temperature (°C)**	**Expected product size (bp)**
CtaCV-1	F-CCA CCA GAC TGG GAC GTA GT R-ACA GCC GTC AAA CCA TTT TC	58	555
CtaCV-2	F-AGA ACA GGG AAC TCC CCA CT R-CGC AAC GTT CAC AAA GAA GA	54	592
CtaCV-3	F-ATG CCT GGT TAC CAA TTT CG R-TCA AGC CAT CGA GTT TTT CC	58	521
CtaCV-4	F-GCA AGA CAC AGC TGG AAA CA R-CTC CAC GAA GCT TTT TAC CG	50	593

## Results and discussion

Since gelatinous organisms are an important component of plankton communities and their blooms negatively affect human activities such as aquaculture, infrastructure, and tourism (Purcell et al., 2007; Purcell, 2012), it is critical that we gain a better understanding of the factors affecting their ecology and regulating bloom dynamics. This research is of increasing importance since a recent study demonstrated increasing trends in gelatinous organisms, including ctenophores, since 1950 in >70% of surveyed large marine ecosystems (Brotz et al., 2012). In addition, there is increasing evidence that anthropogenic influences (eutrophication and overfishing) and global climate change are driving changes in the phenology, magnitude, and geographical extent of gelatinous zooplankton blooms (Mills, 1995; Purcell et al., 2007; Purcell, 2012; Condon et al., 2013). Even in native ranges, blooms appear to be increasing in magnitude and beginning earlier than previously documented (Sullivan et al., 2001; Costello et al., 2006; Condon and Steinberg, 2008; McNamara et al., 2010; Robinson and Graham, 2014).

An important component of understanding ctenophore ecology is characterizing the myriad of other organisms associated with ctenophores, including both parasites and symbionts. An increasing number of studies have shown that ctenophores interact with a diverse range of invertebrates (amphipods, anemones, worms), protozoa (amoeba, ciliates), dinoflagellates, and bacteria; however, to date, no studies have investigated the association of viruses with these organisms. Following recent studies that have discovered a diversity of CRESS-DNA viruses in marine invertebrates, this study surveyed the presence of this viral group in ctenophores. *M. leidyi* (*n* = 127) specimens collected between February and October 2013 as well as *B. ovata* (*n* = 26) specimens collected between April and August 2013 from the Skidaway River Estuary and the Savannah River in coastal Georgia, USA (Supplemental Table 1) were screened for the presence of CRESS-DNA viruses using RCA followed by RE digestion. To minimize the possibility of contamination from food sources, the stomachs were removed from most of the specimens by dissection using sterile techniques, and co-occurring copepods were collected at each sampling event for inclusion in prevalence studies.

A total of 17 circular DNA molecules, ranging in length between 1030 and 2838 nt, were recovered from both *M. leidyi* (*n* = 10) and *B. ovata* (*n* = 2) samples (Table 2). Eight of these molecules encode a Rep and contain a putative *ori* marked by a conserved nonanucleotide motif (NANTATTAC), thus representing CRESS-DNA genomes (**Figure 2**; GenBank accession numbers KT945162-KT945169). The remaining nine circular DNA molecules detected in this study can be divided into several categories: (i) genomes containing a putative *ori* but no identifiable ORFs (*n* = 3); (ii) genomes containing a putative *ori* and identifiable ORFs that lack similarities to known Reps (*n* = 3); (iii) genomes that lack a putative *ori* but have identifiable ORFs that lack or have weak similarities to known Rep proteins (*n* = 3). At this time it is difficult to assess what type of genetic entity these nine circular molecules represent and, thus, they were not further explored.

**Table 2:** **Circular DNA molecules detected in ctenophores, including ctenophore-associated circular virus (CtaCV) genomes and ctenophore-associated circular genomes (CtaCG)**.

**Genome^a^(accession no.)**	**Species^b^**	**Location / Date**	**Genome size (nt)**	**Genome architecture^c^**	**Nonanucleotide motif**
CtaCV-1 (KT945162)	*M. leidyi*	Skidaway River / 9-19-13	1846	Type V	TAGTATTAC
CtaCV-2 (KT945163)	*M. leidyi*	Skidaway River / 3-21-13	1855	Type I	CATTATTAC
CtaCV-3 (KT945164)	M. leidyi^*^	Skidaway River / 4-15-13	1722	Type V	TAGTATTAC
CtaCV-4 (KT945165)	M. leidyi^*^	Skidaway River / 4-3-13	1714	Type II	TAGTATTAC
CtaCG-1 (KT945166)	*M. leidyi*	Skidaway River / 9-19-13	1210	Type VII	CATTATTAC
CtaCG-2 (KT945167)	*B. ovata*	Skidaway River / 7-16-13	1210	Type VII	CATTATTAC
CtaCG-3 (KT945168)	*B. ovata*	Skidaway River / 7-16-13	1223	Type VII	CATTATTAC
CtaCG-4 (KT945169)	*B. ovata*	Skidaway River / 7-16-13	1210	Type VII	TAGTATTAC
I_1192_K_A10	*M. leidyi*	Skidaway River / 8-20-13	2209	(no *ori*)	X
I_1192_K_A4	*M. leidyi*	Skidaway River / 8-20-13	2838	(no *ori*)	X
I_1106_P_H3	*M. leidyi*	Skidaway River / 6-11-13	1030	(no Rep)	TAGTATTAC
I_1106_P_G3	*M. leidyi*	Skidaway River / 6-11-13	1060	(no Rep)	CAGTATTAC
I_1105_P_B7	*M. leidyi*	Skidaway River / 6-6-13	1155	(no Rep)	AATTATTAC
I_1105_P_A8	*M. leidyi*	Skidaway River / 6-6-13	1165	(no *ori*)	X
I_1090_P_H10	*M. leidyi*	Savannah River / 4-8-13	1737	(no ORFs)	TAATATTAC
I_1089_E_4	*M. leidyi*	Skidaway River / 4-15-13	1932	(no ORFs)	GACTATTAC
I_0880_P_C1	M. leidyi^*^	Savannah River / 4-8-13	1731	(no ORFs)	TAATATTAC

a *Only circular DNA molecules representing circular Rep-encoding ssDNA (CRESS-DNA) genomes were named and deposited into GenBank. These molecules include CtaCVs as well as CtaCGs that may represent satellites, genomic components of multipartite viruses, or non-viral mobile genetic elements. Sequences for detected circular DNA molecules not representing CRESS-DNA genomes are provided in Supplemental File 1 with the ID indicated in the first column*.

b The presence of an asterisk (^*^) by the species name highlights samples for which two individuals belonging to the same species that were collected at the same time were pooled for processing.

c *Genome architectures are assigned depending on the presence of an origin of replication (ori), marked by a conserved nonanucleotide motif (NANTATTAC), relative to the Rep-encoding open reading frame (ORF) (Rosario et al., 2012b). In various cases it was not possible to assign an architecture*.

Half of the identified CRESS-DNA genomes contain two major ORFs encoding putative Rep and hypothetical structural proteins, whereas the other four genomes exhibit a single major ORF encoding the Rep. The ORFs encoding hypothetical structural proteins (non-Rep-encoding ORFs) were analyzed for IDP regions. IDP profile analysis revealed that these putative structural proteins exhibited patterns resembling the Type A IDP profiles previously identified in the majority of capsid proteins encoded by known CRESS-DNA viral genomes (Rosario et al., 2015). Therefore, the IDP profiles provide further support to the identified hypothetical structural proteins likely representing capsid proteins.

**Figure 1:**
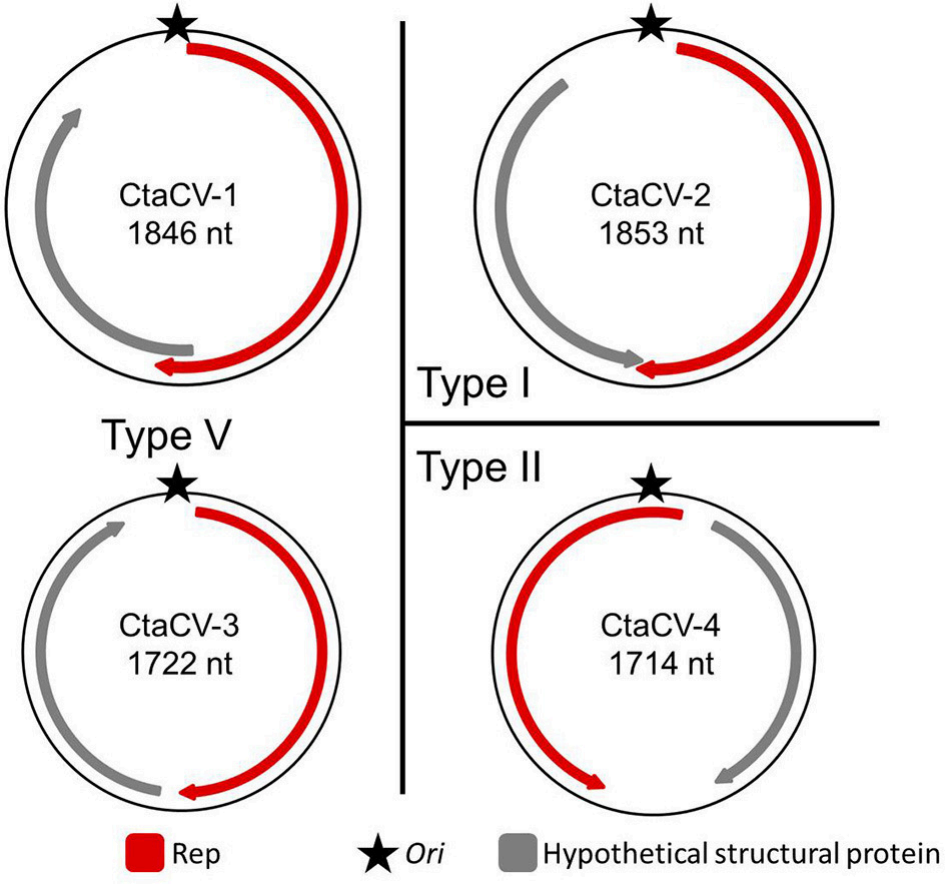
**Genome types of novel ctenophore-associated circular viruses (CtaCVs) identified in this study**. Genome schematics indicate a major ORF encoding the replication initiator protein (Rep; red), a putative origin of replication (*ori*; star) and a second major ORF representing a hypothetical structural protein (gray).

Based on the position of the Rep-encoding ORF relative to the *ori*, the ctenophore-associated CRESS-DNA genomes identified here belong to Types I, II, V, and VII (Figure 1) as defined by Rosario et al. (2012b). Since type VII genomes only exhibit a single ORF, these genomes may represent partial genomes of multipartite viruses, satellite DNA molecules that require helper viruses, or non-viral mobile genetic elements such as plasmids, and therefore cannot be considered viral genomes (Rosario et al., 2012b). Therefore, four ctenophore-associated viruses were identified, named here Ctenophore-associated circular virus (CtaCV)-1, CtaCV-2, CtaCV-3, and CtaCV-4 (Table 2).

**Figure 2:**
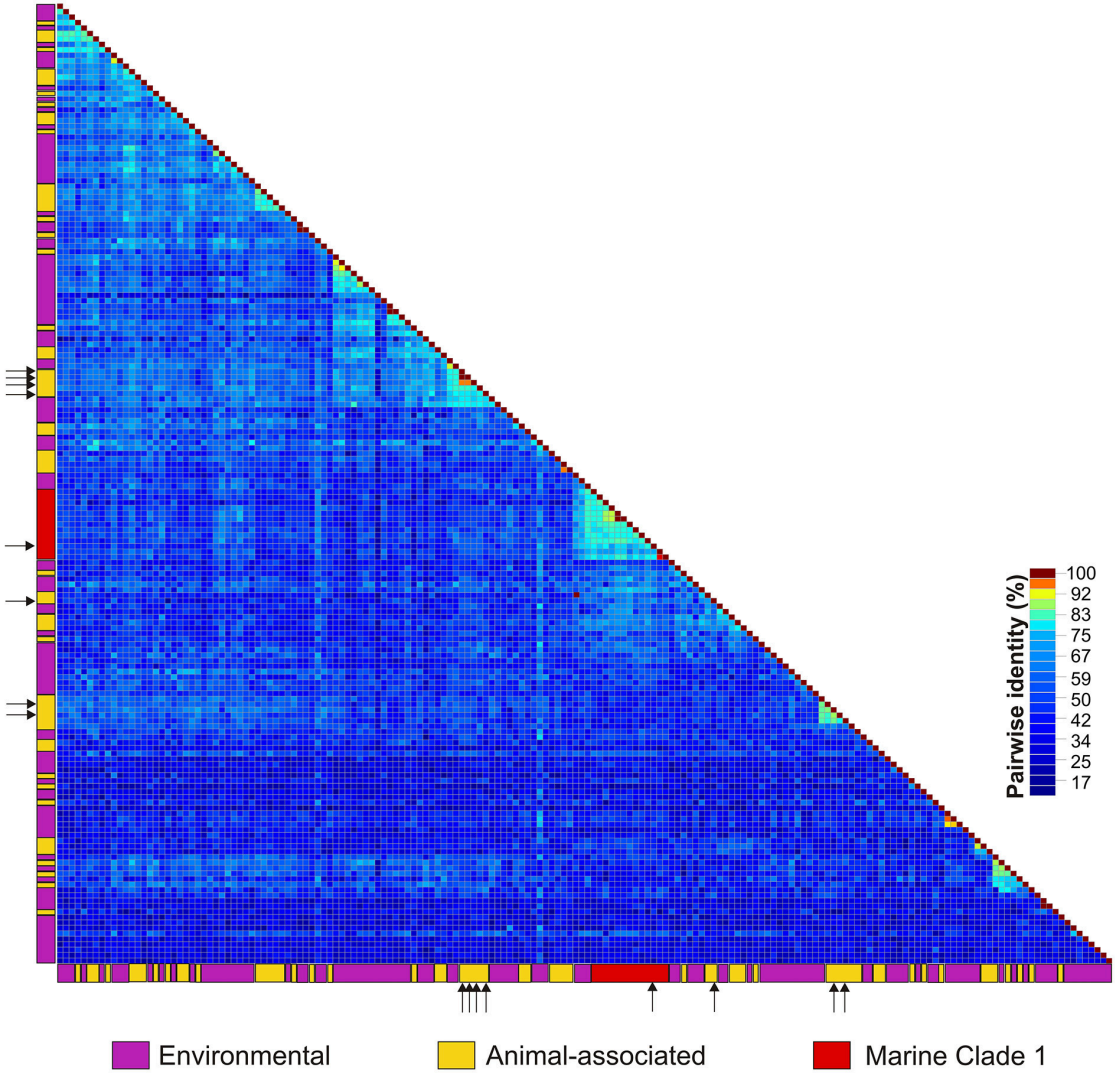
**Heatmap illustrating pairwise amino acid identities of the replication initiator proteins (Rep) from CRESS-DNA genomes identified in animals (yellow), including those reported in this study (arrows), and the marine/estuarine environment (purple)**. Sequences representing the CRESS-DNA Marine Clade 1 identified in Rosario et al. (2015) are highlighted in red.

The identified ctenophore-associated CRESS-DNA genomes were most similar to viral sequences identified through metagenomic surveys of marine or estuarine environments (Supplemental Table 2). Each of the eight genomes was unique as they only shared 54–77% genome-wide pairwise identities. The highest genome-wide identity (77%) was observed between two Type VII genomes identified in *M. leidyi* and *B. ovata* specimens; however, the viral genomes (CtaCV-1 through CtaCV-4) shared less than 60% identity. Comparison of the ctenophore-associated genomes to previously published marine CRESS-DNA genomes in GenBank revealed a high level of divergence. Based on Rep pairwise identity comparisons, the four ctenophore-associated viral genomes share less than 60% identity (range = 37–59%) with previously reported CRESS-DNA genomes indicating that each represents a novel species (Figure 2). The ctenophore-associated CRESS-DNA viruses identified here are as distantly related to each other (Rep pairwise identity range = 22–49%) as they are to viruses identified in other marine organisms and environments even though they were all retrieved from the same species, *M. leidyi*.

Although CRESS-DNA viruses have been reported from various organisms, so far there are no distinct clusters defined by the location, environment, or type of organism from which the viruses were sampled. Furthermore, most of the reported CRESS-DNA viruses represent novel species. Based on Rep pairwise identities (Figure 2), one of the ctenophore-associated viruses identified here, CtaCV-2, belongs to the previously described CRESS-DNA Virus Marine Clade 1 (Rosario et al., 2015). In addition, similar to members of the reported Marine Clade 1, CtaCV-2 exhibits a Type I genome architecture. Marine Clade 1 may represent a novel group at the family level (average Rep pairwise identities of 47%) and includes viruses sampled from a diversity of invertebrates (crustaceans, bivalves, cnidarians, gastropods, mollusks, tunicates, and ctenophores). The diversity and low identities shared among marine CRESS-DNA viruses reported to date indicates that the marine environment is a rich source of CRESS-DNA viruses.

**Figure 3:**
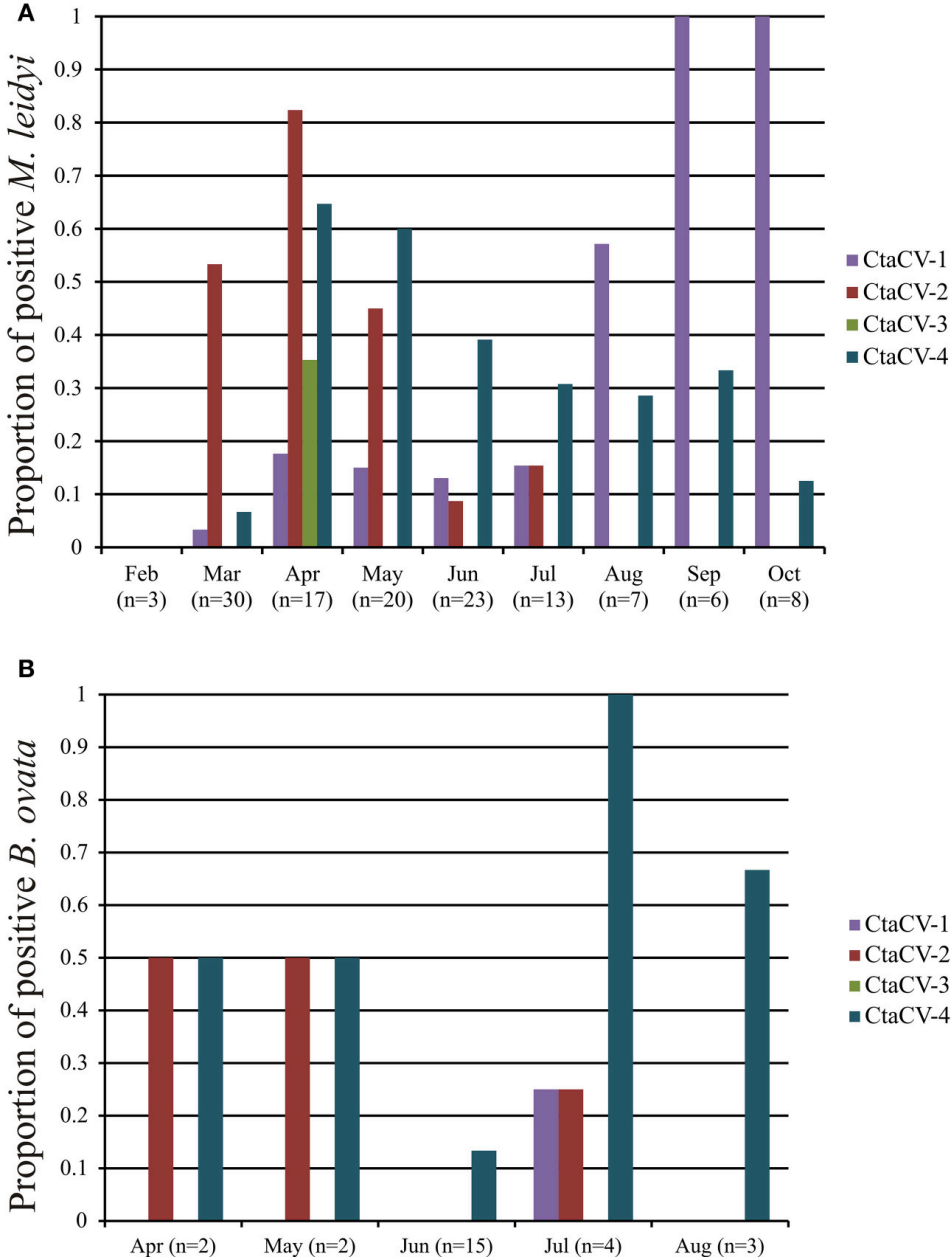
**Prevalence of ctenophore-associated circular virus (CtaCV) genomes in (A) ***M***. ***leidyi*** and (B) ***B. ovata*** collected over time as determined by specific PCR**.

**Figure 4:**
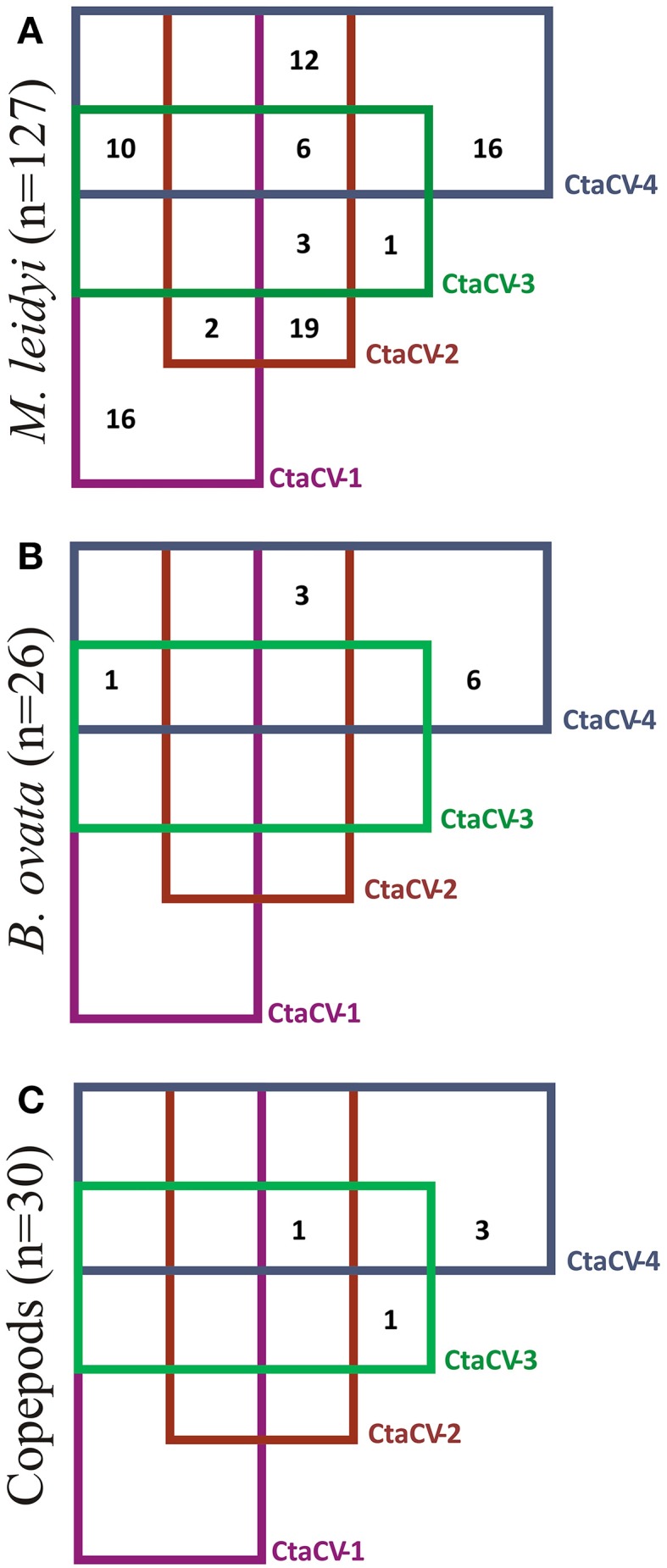
**Venn diagram showing the prevalence of each of the ctenophore-associated circular virus (CtaCV) genomes alone or in combination with other CtaCV genomes in (A) ***M***. ***leidyi***, (B) ***B. ovata***, (C) pools of copepods**.

To further explore the prevalence of the four CRESS-DNA viral genomes discovered in *M. leidyi*, specific PCR primers were designed to amplify a portion of the Rep-encoding gene from each virus (Table 1). RCA products were used as template for the PCR assays, thus providing more sensitivity and a more accurate assessment of viral prevalence than the RCA-RE approach alone. The PCR assays revealed the widespread presence of these viruses in individual *M. leidyi* specimens collected between February and October 2013, with 66% of the 127 specimens testing positive for one or more of the viruses (Table 3; Supplemental Table 1). In addition, temporal trends in dominant viral types were evident through the time-series (Figure 3A). CtaCV-1, which was originally discovered in a sample from September 2013, was detected in *M. leidyi* specimens collected between March and October. However, CtaCV-1 was present in < 20% of individuals collected before August and >50% of individuals between August and October indicating that this virus became more prevalent in the fall. All 14 *M. leidyi* specimens collected in September and October tested positive for this virus. Seasonal trends were also observed for the other viruses. CtaCV-2, which was originally discovered in a specimen from March 2013, was only detected in samples collected between March and July 2013, with a peak in prevalence in April when this virus was present in over 80% of *M. leidyi* specimens. CtaCV-3 and CtaCV-4 were both originally identified in samples from April 2013, which was also the month of peak prevalence for each of these viruses (35% for CtaCV-3 and 65% for CtaCV-4). These results indicate that the RCA-RE method detected these viruses in individuals when the viruses were most prevalent in the population. The PCR data suggest that the ctenophore-associated CRESS-DNA viruses exhibit seasonal variation; however, some viruses may be present in the ctenophore population year-round since CtaCV-1 and CtaCV-4 were detected each month between March and October. Despite the persistence of these two viruses throughout the sampling period, temporal patterns were present with CtaCV-4 dominating in the spring/early summer and CtaCV-1 dominating in the fall. Additionally, several *M. leidyi* specimens tested positive for multiple viruses, with 17 individuals positive for two viruses and 16 individuals positive for three viruses (Figure 4A). Efforts to link ctenophore length and volume with viral presence did not reveal any trends.

**Table 3:** **Prevalence of ctenophore-associated circular viruses (CtaCVs) detected in individual ctenophores and pooled copepod samples**.

**Sample**	**Total number tested**	**Positive samples (%)**
*Mnemiopsis leidyi*	127	66
*Beroe ovata*	26	38
Copepods	30^*^	17

* *Copepods were tested in batches of approximately 20 specimens, which were dominated by late copepodites and adults of Acartia tonsa*.

The PCR assays were also used to test specimens of *B. ovata*, a predator of *M. leidyi*, for the presence of the four CRESS-DNA viruses. Thirty-eight percent of the *B. ovata* samples (*n* = 26) collected between April and August 2013 tested positive for one or more of the viruses (Table 3; Supplemental Table 1). Although the total number of *B. ovata* specimens available for testing was much lower than that of *M. leidyi*, some trends in viral prevalence can be observed from the temporal data (Figure 3B). CtaCV-4 was the most prevalent virus in *B. ovata* and, similar to *M. leidyi*, was found in each month where samples were collected. All of the positive *B. ovata* samples (*n* = 10) contained CtaCV-4, with three of those also containing CtaCV-2 and one also containing CtaCV-1 (Figure 4B). CtaCV-3 was never identified in *B. ovata*, and CtaCV-1 was only found in a single specimen. Since *B. ovata* comb rows (as opposed to whole specimens) were processed, it is unlikely that the viruses originated from their prey; however, both of the most prevalent viruses detected in *M. leidyi* (CtaCV-2 and CtaCV-4, each detected in 43% of specimens) were also detected to a lesser extent in *B. ovata*.

Finally, since CRESS-DNA viruses have been detected in copepods (Dunlap et al., 2013) and these planktonic organisms are a major food source for *M. leidyi*, copepods collected concurrently with the ctenophore samples were tested for the ctenophore-associated viruses. The copepods were screened in batches of approximately 20 individuals using PCR. Only five of the 30 batches of copepods tested positive for any of the viruses (Table 3; Supplemental Table 1), with three positive for only CtaCV-4, one positive for only CtaCV-3, and one positive for CtaCV-2, CtaCV-3, and CtaCV-4 (Figure 4C). Notably one of the most persistent viruses in *M. leidyi* specimens, CtaCV-1, was never detected in copepod samples. The much lower prevalence of these viruses in pooled batches of copepods (17%) compared to individual *M. leidyi* (66%), combined with the removal of the ctenophore stomachs before processing, suggest that these viruses likely do not originate from copepod prey.

Given the importance of gelatinous zooplankton for global biogeochemistry and the direct impacts of blooms on ecosystem dynamics and human activities, examining the presence of viruses in ctenophores addresses an important knowledge gap. A great deal of previous research has examined the conditions that favor ctenophore growth and reproductive success (Purcell et al., 2001; Costello et al., 2012; McNamara et al., 2014; Robinson and Graham, 2014; Gambill et al., 2015; Jaspers et al., 2015); however, significantly less attention has been given to factors that contribute to bloom demise (Rathjen et al., 2012). Both physical (low salinity, thermal stress, intertidal stranding) and biological (parasite infestation, disease, senescence following spawning, predation, food limitation) parameters have been frequently cited in jellyfish bloom demise (Pitt et al., 2014); however, no studies have directly investigated ctenophore bloom termination. While their roles in ctenophore ecology and mortality remain completely unknown, this study provides the first evidence of viruses associated with ctenophores. Genome sequence information from the ctenophore-associated viruses will enable quantitative studies addressing their roles, if any, in ctenophore bloom development and demise.

The discovery of novel CRESS-DNA viruses in undersampled marine taxa, such as ctenophores, continues to expand our rapidly-changing perspective on the potential host range and prevalence of these viruses. Along with recent evidence compiling the widespread nature of diverse CRESS-DNA viruses in aquatic invertebrates (Hewson et al., 2013a,b; Ng et al., 2013; Pham et al., 2014; Soffer et al., 2014; Dayaram et al., 2015; Rosario et al., 2015) and environments (López-Bueno et al., 2009; Rosario et al., 2009; Labonté and Suttle, 2013; Roux et al., 2013; Yoshida et al., 2013; Zawar-Reza et al., 2014), this study supports a role for these viruses in aquatic food webs and biogeochemical cycling. Additionally, as knowledge expands regarding CRESS-DNA virus diversity in both vertebrates and invertebrates, analysis of viruses in ctenophores may provide interesting evolutionary insights since genomic sequencing of the complete *M. leidyi* genome suggests that ctenophores may be the earliest diverging animal lineage (Ryan et al., 2013).

Finally, since blooms of gelatinous organisms have significant effects on many aspects of ecosystem structure, biogeochemical cycling, and human interactions with coastal environments, future work needs to document the presence of virus particles within ctenophore tissues via electron microscopy (e.g., Dunlap et al., 2013; Soffer et al., 2014) and should strive to determine the tissue specificity, biogeography, and ecological roles of these ctenophore-associated viruses. The persistence and high prevalence of some of the ctenophore-associated CRESS-DNA viruses, combined with the importance of ctenophores in aquatic ecosystems and their expanding geographic ranges, make ctenophores an ideal system for studies of the potential impacts of CRESS-DNA viruses on the feeding, reproduction, behavior, and health of marine invertebrates.

## Author contributions

MB, KR, JN conceived the study. LB, SB collected samples and metadata. BB, PJ, RH, SF, CG, AW, DG processed samples and performed all lab analyses. MB, KR analyzed data and wrote the manuscript. All authors edited and approved the final manuscript.

### Conflict of interest statement

The authors declare that the research was conducted in the absence of any commercial or financial relationships that could be construed as a potential conflict of interest.
