# Supplementary material for: Discovery, Prevalence, and Persistence of Novel Circular Single-Stranded DNA Viruses in the Ctenophores Mnemiopsis leidyi and Beroe ovata
Source: Front Microbiol. 2015 Dec 18;6:1427. doi: 10.3389/fmicb.2015.01427 (PMC4683175; doi:10.3389/fmicb.2015.01427)
Supplement: Supplementary file 2 [file Table2.docx]

| **Genome** | **Rep-encoding ORF**  **(accession no.)** | **ORF encoding hypothetical structural protein**  **(accession no.)** |
| --- | --- | --- |
| CtaCV-1 | Avon-Heathcote Estuary associated circular virus 18 (YP_009126920) | Sewage-associated circular DNA virus-16 (YP_009116895) |
| CtaCV-2 | Avon-Heathcote Estuary associated circular virus 24 (YP_009126936) | McMurdo Ice Shelf pond-associated circular DNA virus-1 (YP_009047127) |
| CtaCV-3 | Avon-Heathcote Estuary associated circular virus 18 (YP_009126920) | Odonata-associated circular virus-3 (AJD07504) |
| CtaCV-4 | Picobiliphyte sp. MS584-5 nanovirus (AEI54346) | Uncultured marine virus (AGA18274) |
| CtaCG-1 | Uncultured marine virus (AGA18366) | X |
| CtaCG-2 | Uncultured marine virus (AGA18366) | X |
| CtaCG-3 | Uncultured marine virus (AGA18366) | X |
| CtaCG-4 | Uncultured marine virus (AGA18366) | X |

Supplemental Table 2. Top BLAST matches for ctenophore associated viruses (CtaCVs) and genomes (CtaCGs) by open reading frame (ORF).
